# Difference in Body Weight at Breeding Affects Reproductive Performance in Replacement Beef Heifers and Carries Consequences to Next Generation Heifers

**DOI:** 10.3390/ani11102800

**Published:** 2021-09-26

**Authors:** Ramanathan K. Kasimanickam, Vanmathy R. Kasimanickam, Madison L. McCann

**Affiliations:** 1 Department of Veterinary Clinical Sciences, College of Veterinary Medicine, Washington State University, Pullman, WA 99164, USA; vkasiman@wsu.edu (V.R.K.); madison.mccann@wsu.edu (M.L.M.); 2 AARVEE Animal Biotech LLC, Corvallis, OR 97333, USA

**Keywords:** beef heifers, prebreeding, nutrition, body weight, generations, reproductive performance

## Abstract

**Simple Summary:**

The effect of prebreeding feeding to attain 55% vs. 65% of mature cow body weight (MBW; 545 kg) at breeding on the reproductive performance of beef heifers and its offspring was investigated. Angus-cross dam heifers from weaning were randomly fed to attain 55% (*n* = 1622) vs. 65% (*n* = 1578) of MBW and off-spring (F1) heifers born to dam heifers in both 55% (*n* = 1285) vs. 65% (*n* = 1324) groups were fed to attain 65% of MBW. Results showed that restricted feeding negatively affected puberty, breeding season pregnancy and 21-day calving rates. F1 generation heifers that were fed normal diets but were born to dams that were fed restricted diets also had reduced puberty, breeding season pregnancy and 21-day calving rates. In conclusion, restricted feeding during the prebreeding period of dam heifers reduced post-pubertal fertility and fertility of their heifer offspring that were fed normal prebreeding diets.

**Abstract:**

Nutrition imprinting carries consequences across generations. The effect of 55% vs. 65% of mature cow body weight (MBW; 545 kg) at breeding on the reproductive performance of heifers and their offspring was investigated. Angus-cross dam heifers were randomly fed to attain 55% (*n* = 1622) vs. 65% (*n* = 1578) of MBW, and offspring (F1) heifers born to dam heifers [55% (*n* = 1285) vs. 65% (*n* = 1324)] were fed to attain 65% of MBW. Bodyweight and reproductive indices were recorded throughout the study. In dam heifers, puberty (44% vs. 53%), breeding season pregnancy (86.4% vs. 90.6%) and 21-day calving rates (55.2% vs. 65.4%) did vary, but dystocia rate (8.7% vs. 9.0%) did not differ between 55% and 65% MBW groups. Puberty (49.2% vs. 58.2%), breeding season pregnancy (87.2% vs. 92.8%) and 21-day calving rates (53.8% vs. 64.1%) did differ (*p* < 0.05), but dystocia rate (8.4 vs. 9.2%) did not differ between F1 heifer groups. In conclusion, 55% of MBW at breeding negatively affected the reproductive performance of heifers and its offspring heifers. The recommendation is to feed heifers a balanced diet to reach 65% of MBW at breeding with consideration of production traits.

## 1. Introduction

Production performance of replacement beef heifers born earlier in the calving season was greater than those that were born later in the calving season [1,2,3]. Many key factors play a vital role, including heifers that were born to genetically superior parents, were born early in the calving season and attained puberty early. In addition, greater lifetime productivity of beef replacement heifers can be expected if they have first calving by 2 years of age. To achieve this, heifers need to be bred and conceived at 15 months of age to calve at 24 months of age [1,3]. 

Age at puberty in beef heifers is determined by genetics, nutrition and environment [4,5,6,7]. Adequate growth in replacement heifers is necessary for reaching the pubertal status prior to the breeding period [7]. Body weight (BW) is a primary determinant of puberty attainment in heifers. Beef heifers usually achieve puberty when they reach 55% to 60% of mature BW [8]. There are several different strategies for feeding heifers from post-weaning to pre-breeding, all of which depend on the average daily gain being appropriately calculated to reach the desired percentage of mature cow BW (MBW) at breeding. Realistically, the heifers should be fed a high-energy diet for a minimum of 80 days prior to reaching the target of 65% of MBW at breeding [8]. 

Heifers with a faster growing weight (higher average daily gain) prior to weaning reach puberty faster [5]. In heifers, by altering the rates of gain and the total percentage of mature BW achieved prior to breeding, it is possible to influence the performance of their calves and subsequent pregnancy rate [7]. Additionally, first-generation heifer calves retained for replacement regardless of their feeding strategy can potentially carry their reproductive traits set by their dam’s percentage of MBW at breeding [3,9]. Nutritional imprinting refers to the epigenetic programming during the prenatal and neonatal periods by altering a dam’s nutrition, which can have significant consequences later on in an offspring’s life [10]. Several factors that were investigated involved in nutritional imprinting of heifers including cow’s nutrition during pregnancy, milk production of the cow, birth weights of calves born and weights of calves at weaning, which can be correlated to the percentage of mature weight of a dam at first breeding [4,8,9]. In addition, managing nutrient supply to match demand is critical for sustainable and efficient livestock production, which necessitates careful preparation for a given situation.

The object of this study was to investigate the effect of percentage of mature BW, 55% vs. 65%, at the time of breeding on the reproductive performance of replacement heifers and their first-generation (F1) heifers.

## 2. Materials and Methods

### 2.1. Heifers

Angus-cross replacement beef heifers (*n* = 3200) from 12 farms during the 2015 to 2019 spring breeding season were included. Heifers’ birthweight (using Calfscale^®^ tape; the tape was placed around the coronary band of either front hoof and tightened and then the circumference and the birthweight in pounds was recorded) [11] and history of assisted delivery (Score 0 to 4 points: 0, unassisted; 1 and 2, easy pull; 3, hard pull; 4, C-section (excluded)) [12,13] were recorded immediately after parturition. To avoid selection bias, all heifers born early (first 21 days) in the calving season were included. Selected heifers were free of respiratory and digestive disorders and were subjected to routine herd health management.

Heifers that met selection criteria were subjected to feeding strategies to meet 55% vs. 65% mature BW at breeding (average cow mature weight is 1200 lbs. (545 kg); a 65% MBW target of 780 lbs. (355 kg) and a 55% MBW target of 660 lbs. (300 kg) were considered). They were randomly fed to attain 55% (*n* = 1622) and 65% (*n* = 1578) of MBW. Heifers were developed in the ranch with or without irrigated pasture. When heifers were in a period of nutritional stress, such as when grazing poor-quality winter forage, protein supplementation was instigated. Heifers were weighed at weaning and periodically thereafter. The average daily gain was calculated using the interval (days) between weaning and breeding and differences in weaning and breeding BW. The average daily gain varied from 1.1 to 1.5 lbs./day for the 55% MBW group and 1.7 to 2.0 lbs./day for the 65% MBW group. The feed was mixture of hay and grain corn/mixed grain (or) protein supplement. To meet the target weight, it was calculated that those heifers in the 55% MBW group needed 12.4 lbs. DM, 60% TDN and 10% CP, and heifers in the 65% MBW group needed 12.7 lbs. DM, 67% TDN and 12% CP. First-generation replacement heifers were fed to attain 65% MBW at breeding. As the gain from weaning to breeding will not be steady, heifer groups were weighed (weigh bridge) periodically to adjust feeding. Examples of two hay analysis results are given in Supplementary Tables S1 and S2. 

### 2.2. First-Generation Heifers

F1 heifers (*n* = 2609) born during the first 3 weeks to dam heifers in the 55% (*n* = 1285) and 65% (*n* = 1324) MBW treatment groups were included to investigate the effect of dams’ BW at breeding on their daughters’ performance. Similar to their dams, heifers’ birth-weight and history of assisted delivery were recorded immediately after parturition. To avoid selection bias, all heifers born early (first 21 days) in the calving season were included. Selected heifers were free of respiratory and digestive disorders and were subjected to routine herd health management. Progeny heifers were fed to reach 65% mature BW at breeding irrespective of the dam’s weight group. Breeding management was similar to their dams. The heifers in both groups were fed similarly to meet NRC recommendations during pregnancy.

### 2.3. Breeding

The heifers were weighed, palpated transrectally and assigned a reproductive tract score (1 to 5; 1—immature, acyclic; 5—mature, cyclic [14]. Freemartin heifers were excluded from the experiment. Heifers with corpus luteum were considered as pubertal [15] and received a pre-breeding vaccine. Heifers were penned with natural service sires (bull-to-cow ratio 1:25) in an 85-day breeding season. Bodyweight and reproductive indices were recorded from time to time during the study period.

### 2.4. Pregnancy Diagnosis

Pregnancy diagnosis was performed approximately 30 days after the end of breeding season, using transrectal ultrasonography (Sonoscape S8 with 5 MHz linear-array transducer, Universal Imaging, Bothell, WA, USA). Positive pregnancy diagnosis was based on the visualization of a viable embryo/fetus.

### 2.5. Statistical Analyses

Data were analyzed using a statistical software program (SAS Version 9.4, SAS Institute, Cary, NC, USA). For all analyses, *p* ≤ 0.05 was considered significant.

Differences between treatments in mean BW was determined using ANOVA (PROC GLM). Bartlett test was used to assess the homogeneity of variance. Because variances for means were heterogeneous, log10-transformed data were analyzed, but non-transformed values were reported. PROC GLIMMIX of SAS was used to examine treatment (55% vs. 65% MBW) effects on percentages of puberty, pregnancy, calving difficulty and 21-day calving. Locations nested in years were used as random effects.

## 3. Results

Mean body weight (kg) at weaning and at breeding and average daily gain for dam heifers across locations and years are given in Supplementary Tables S3 and S4, respectively.

### 3.1. Dam Heifers

The weaning weight did not differ between dam heifers in the 55% and 65% MBW groups, 232 vs. 235 kg, respectively (*p* > 0.1; Table 1). Target mature BW at breeding differed between dam heifers in the 55% and 65% BW groups, i.e., 305 and 349 kg and 56% and 64% of mature BW, respectively. Similarly, pre-calving BW differed between heifers in the 55% and 65% MBW groups, i.e., 436 and 458 kg and 80% and 84% of mature BW, respectively. The puberty rate percentages, breeding season pregnancy rate and 21-day calving rate differed, but the calving difficulty rate did not differ between dam heifers in the 55% and 65% BW groups (Table 1).

### 3.2. First-Generation Heifers

Birth weight did not differ (*p* > 0.1), but weaning weight differed (*p* < 0.01) between F1 heifers born to dams in the 55% and 65% MBW groups (Table 2). Prebreeding target BW differed between F1 heifers born to dams in the 55% and 65% MBW groups, i.e., 343 and 355 kg and 63% and 65% of mature BW, respectively (*p* < 0.05). Similarly, pre-calving mature BW differed between F1 heifers born to dams in the 55% and 65% MBW groups, i.e., 442 and 469 kg and 81% and 86% of mature BW, respectively (*p* < 0.01). Puberty rate percentages, breeding season pregnancy rate and 21-day calving rate differed, and calving difficulty rate did not differ between F1 generation heifers born to dams in the 55% and 65% MBW groups (Table 2).

## 4. Discussion

Early attainment of puberty is associated with greater pregnancy rates [5]. Conception early in the breeding season is important for maximizing lifetime reproductive efficiency, where replacement heifers are bred to calve at 24 months of age [3]. It is widely accepted that plane of nutrition from weaning to the onset of the breeding season can affect age at puberty [4,16,17]. The results from Gassard et al. [18,19,20,21] showed that puberty can be consistently induced by initiation of feeding a high-energy diet in beef heifers. Feeding a high-energy diet resulted in 81.6% (31/38) heifers attaining puberty at 265 days compared with control diet, which resulted in 21.1% (8/38) heifers attaining puberty at 348 days. Conventionally, the recommendation has been that replacement heifers must be fed to attain 65% of their expected mature BW by the onset of the breeding season to achieve acceptable reproductive performance. However, several studies suggest that different BW at breeding could be targeted without affecting reproductive performance [3,17]. Recent research has suggested that the development of heifers to 55% of mature BW may present an economic advantage over developing heifers to 65% of mature BW by the time of breeding [22]. Even though disagreement exists as to the ideal target weight for heifers at breeding, nutritional management during this phase is undeniably crucial to breeding success. 

Previous studies investigated postweaning dietary management of heifers to attain different target BWs by the time of breeding and its effect on reproductive performance [23]. Other studies included beef cows to investigate the effect of dam nutrition on growth and reproductive performance of female progenies [24]. These two concepts were studied separately. In the present study, different dietary management of heifers that were fed to attain 65% vs. 55% of mature BW at the time of breeding was utilized to examine the effect of divergence in bodyweight on the onset of puberty and subsequent pregnancy in heifers and impact of dams postweaning growth rate and BW at the time of breeding on the reproductive performance of their heifer offspring that were fed normal diet to attain 65% mature BW at the time of breeding [25]. This comprehensive multifactorial study investigated the interactive effects of prepubertal nutrition on pubertal status by the time of breeding as well as the latent effects of these factors on post-pubertal fertility of the current generation and of their heifer offspring.

Pregnancy rate within a defined breeding period is dependent on the percentage of heifers that attained puberty at the beginning of the breeding season [25]. This finding led to the development of target bodyweight percentage guidelines, in which it was recommended that replacement heifers should be 60–65% of their estimated mature BW by the onset of the breeding season to ensure that puberty had occurred in a large proportion of the replacement heifers before initiation of breeding [26].

Mature BW is an arbitrary trait without considering the variation in cow BW within a breed and at various stages of the production cycle. Breed-type specific target BW of heifers at different phases of their prepubertal growth trajectory is a more useful recommendation. For example, BW at puberty ranges from 288 to 350 kg and 308 to 340 kg in Angus- and Limousine-sired heifers, respectively [22,27]. These BW ranges are less than those in the present study, for both breeds, and may have reflected a shift in North America towards calving heifers at 2 as opposed to 3 years of age with concomitant positive selection pressure for decreased age at puberty [22,27]. Albeit that there are positive aspects of enhanced postweaning BW gain, the results of several studies indicate that there are some flexibilities in how target BW is attained [3,17], which, in turn, could be exploited to decrease the overall cost of rearing replacement heifers by feeding them to attain lesser MBW at breeding without compromising their performance [22]. However, in the present study, more heifers in the 65% MBW group were pubertal at the beginning of the breeding season and had a greater pregnancy rate, which is consistent with results in previous reports [8,23,25,28]. In the current study, 9% fewer dam heifers were pubertal in the 55% MBW group compared with the 65% MBW group. The differences observed in the current study was similar to 8% (fed to appetite, 100% vs. restricted feeding, 80%) [23] and 11% (50 vs. 60% MBW) [8] differences in pubertal status, as a consequence of feeding different quality diets to attain different target BW at breeding. Martin et al. (2008) reported that 17% fewer heifers were pubertal when fed to attain a target bodyweight of 51% MBW compared with 57% MBW at the time of breeding, indicating that greater restriction may have a greater effect on puberty [29]. Most importantly, 11.2% fewer F1 heifers born to dams in the 55% MBW group were pubertal compared to F1 heifers born to dams in the 65% MBW group. 

Induction of puberty with hormone manipulation, especially with progesterone-based programs, resulted in acceptable pregnancy during the subsequent breeding season. Hormonal induction of puberty is most effective in heifers that are approaching their spontaneous occurrence of puberty. Additionally, there are age limits before which it is not possible to effectively induce the first ovulation with hormonal manipulation [30]. Notably, these approaches are not a substitute for physiological development and nutritional management. It should be noted that heifer development implemented with proper nutrition has consequences on the reproductive performance beyond first breeding. 

Breeding heifers early in the breeding season has a predominant effect on herd reproduction and economic efficiency. Beef heifers that were pregnant early in the initial breeding season and calved by 24 months of age had a greater probability of conceiving early as primiparous cows, greater lifetime production and tended to calve earlier in subsequent years compared with females that conceived later in the first breeding season [16]. Roberts et al. (2017) reported a greater overall pregnancy rate and a greater percentage calving in the first 21 days of calving season for heifers that were pubertal at the start of breeding compared to those that were not [5]. In the present study, heifers in the 65% MBW group had a greater pregnancy rate during the second breeding season as 2-year-olds. It should be noted that these pregnant heifers calved early compared with pregnant heifers in 55% MBW group. 

In the current study, a greater reproductive performance was observed for F1 heifers born to dam heifers in the 65% MBW group. Maternal nutrition before and during pregnancy may lead to permanent changes in the fetal genome and to long-lasting effects on the offspring phenotype. Manipulating offspring performance through developmental programming via nutritional imprinting is a recently proposed nutritional method to change the potential performance of the subsequent generation in domestic animals. Cushman et al. (2012) showed that heifers born to mature cows fed 125% nutritional maintenance requirements during late gestation conceived earlier in their first breeding season than those born to cows fed either 75% or 100% of nutritional maintenance requirements during that period [24]. Furthermore, a greater number of heifers born to cows that grazed winter pasture and supplemented with crude protein were pubertal at the beginning of the breeding season [31] and had greater pregnancy rates during the first breeding [26] compared to heifers born to cows that were not supplemented with crude protein. Collectively, these results provide evidence that maternal nutrition can impact heifer progeny reproductive performance. It should also be noted that individual component of the maternal diet could impact the offspring performance differently. For instance, heifers born to dams fed diets to attain 55% of mature BW with higher amount of protein supplementation can perform equally to dams fed diets to attain 65% of mature BW with a conventional amount of protein supplementation.

While supporting the concept that growth rate affects the attainment of puberty, restricted nutrition may facilitate retention of heifers with an inherently delayed pattern of sexual development [5]. As noted in the present and other studies [8,27,28], restricted feeding resulted in fewer pubertal heifers, up to 17% at the time of breeding. It should be noted that selection pressure against the later-maturing heifers may be achieved by retaining heifers born earlier in the calving season or reducing the breeding season length [5]. In the current study, selected heifers and their heifer progeny were born during the first 21 days of the calving season. Thus, the observed reduced performance for heifers in the 55% MBW group and their offspring evidently would not have resulted from a selection pressure for late-maturing heifers. In addition, the application of a restricted feeding strategy over the years in a beef operation may have led to the selection of heifers genetically predisposed to delayed sexual development. It is also plausible that implementing feeding strategies to increase the proportion of heifers that achieve puberty before the first breeding could also affect other characteristics or genetics in a herd and this outcome should not be ignored [4]. 

In the current study, the reproductive performance of F1 heifers born to dams in the 55% MBW group was inferior to F1 heifers born to dams in the 65% MBW group. The results showed the latent effect of restricted prepubertal feeding across generations. It should be noted that the dam heifers were fed to meet NRC recommendations during pregnancy in the present study. Dam heifers in the 55% vs. 65% MBW groups had 80% vs. 86% of mature BW at pre-calving, respectively. The growth rate from breeding to calving for dam heifers in the 55% MBW group was 24% (80–56) compared to 20% (84–64) for dam heifers in the 65% MBW group. In theory, considering the four percentage point lesser growth rate for dam heifers in the 65% BW group, they might have received fewer nutrients during pregnancy compared to dam heifers in the 55% BW group. Maternal undernutrition during pregnancy can alter reproductive function of the offspring [10,32,33,34,35]. Considering nutritional imprinting of the fetus that developed in dams with a four percentage point lesser growth rate during pregnancy in the 65% mature BW group, it is expected that the reproductive performance of F1 heifers should have been inferior. Interestingly, the results in the present study showed that F1 heifers developed in dams in the 65% MBW group had improved reproductive performance. It is plausible that not only maternal undernutrition during pregnancy, but also maternal undernutrition before pregnancy or during early postnatal life can have a profound negative effect on the reproductive function of the offspring. With that in mind, selection and management of replacement beef heifers should be performed involving vital decisions that should not affect the overall productivity of the herd [16]. Collectively, a balanced approach between targeted genetic selection for a younger age at puberty together with appropriate nutritional management is warranted.

Economically, a beef cattle operation must weigh between the costs, including feeding heifers with reproductive performance and losses obtained from poor performance later in their life and/or longevity. Therefore, while reproductive performance, longevity and nutritional imprinting are beneficial for the productivity of the heifer, it is imperative to consider the cost–benefit analysis of feeding strategies. Although the reproductive performance of 55% versus 62% mature BW heifers is not different [5,9,25], the economic analysis revealed a reduction in the developing cost of 55% versus 62% mature BW heifers [5,22,27]. However, these studies failed to assess performance of heifers later in life and especially the performance of their offspring. The ideal level of nutritional intervention strategies, and age at puberty and first breeding resulting in optimal economic and reproductive outcome, is not consistent between breeds, production systems and regions [16]. Use of knowledge and technologies, and evaluation of outcomes are necessary to determine the optimal approach in a given situation.

## 5. Conclusions

In conclusion, 55% of mature BW at breeding negatively affected the reproductive performance of the replacement heifers and their offspring compared to traditional 65% of mature BW at breeding. Beef cattle producers are advised to feed heifers to attain 65% target BW at breeding. However, feeding heifers a balanced diet with consideration of production traits is warranted.

## Figures and Tables

**Table 1:** Performance of replacement heifers with 55% vs. 65% mature body weight (BW) at breeding.

Parameters	55% MBW	65% MBW	*p* Value
*n*	1622	1578	-
Weaning BW, kg	236 ± 6.2	234 ± 5.6	*p* > 0.1
Prebreeding BW, kg	305 ± 7.1	349 ± 8.2	*p* < 0.01 *
Prebreeding % mature BW, %	56	64	*p* < 0.05 ^‡^
Pubertal rate, %	44.0 (714/1622)	53.0 (837/1578)	*p* < 0.0001 ^‡^
Pregnancy rate, %	86.4 (1401/1622)	90.6 (1429/1622)	*p* < 0.0002 ^‡^
Pre-calving BW, kg	436 ± 9.1	458 ± 7.9	*p* < 0.01 *
Pre-calving mature BW, % ^†^	80	84	*p* < 0.05 ^‡^
21-day calving rate, %	55.2 (773/1401)	65.4 (934/1429)	*p* < 0.0001 ^‡^
Calving difficulty score	1.0	1.3	*p* > 0.1
Calving difficulty, %	8.7 (122/1401)	9.0 (129/1429)	*p* > 0.1
Easy pull, %	5.9 (83/1401)	5.4 (78/1429)	*p* > 0.1
Hard pull, %	2.8 (39/1401)	3.6 (51/1429)	*p* > 0.1
Second pregnancy rate	84.6 (1110/1312)	93.2 (1226/1316)	*p* < 0.0001 ^‡^

Mature BW = 545 kg (1200 lbs.); * PROC GLM; ^‡^ PROC GLIMMIX; Covariance parameter estimates: Location (year), 0.01229 ± 0.00432; Residual, 0.0865 ± 0.00439; Fit statistics—BIC = 769.28; −2 Res log likelihood = 764.91; Benchmark: ^†^ Breeding season pregnancy rate = 90%; Prebreeding BW (%) = 65% of mature BW; Pre-calving BW = 85% of mature BW. Calculations: % Puberty at the beginning of breeding season = Number of heifers cycling/Total number of heifers. % Pregnancy rate = Number of heifers pregnant/total number. Note: Only heifer calves born in the first 21 of days of the calving season were selected for the study.

**Table 2:** Performance of first-generation replacement heifers born to dams with 55% vs. 65% mature body weight (BW) at breeding.

Parameters	Dam MBW 55	Dam MBW 65	*p* Value
*n*	1285	1324	-
Birth weight, kg	34.1 ± 3.3	35.6 ± 3.7	*p* > 0.1
First calf weaning BW, kg	226 ± 4.3	239 ± 3.4	*p* < 0.01 *
Prebreeding BW, kg	343 ± 5.2	355 ± 6.1	*p* < 0.01 *
Prebreeding mature BW, % ^†^	63	65	*p* > 0.1
Pubertal rate, %	49.2 (632/1285)	60.8 (805/1324)	*p* < 0.0001 ^‡^
Breeding season pregnancy rate, % ^†^	87.2 (1121/1285)	92.8 (1229/1324)	*p* < 0.0001 ^‡^
Pre-calving BW, (kg)	442 ± 7.3	469 ± 8.5	*p* < 0.01 *
Pre-calving mature BW, % ^†^	81	86	*p* < 0.05 ^‡^
21-day calving rate, %	53.8 (603/1121)	64.1 (188/293)	*p* < 0.05 ^‡^
First calf BW, kg	34.9 ± 4.2	36.8 ± 4.3	*p* > 0.1
Calving difficulty score	1.1	1.3	*p* > 0.1
Calving difficulty, %	8.4 (94/1121)	9.2 (113/1229)	*p* > 0.1
Easy pull, %	5.3 (59/1121)	6.5 (80/1229)	*p* > 0.1
Hard pull, %	3.1 (35/1121)	2.7 (33/1229)	*p* > 0.1
First calf weaning BW, kg	228 ± 6.4	254 ± 5.8	*p* < 0.001 *
Second season pregnancy rate, % ^†^	83.7 (946/1130)	92.7 (1116/1204)	*p* < 0.001 ^‡^

Mature BW = 545 kg (1200 lbs.). * Proc GLM; ^‡^ PROC GLIMMIX of SAS version 9.4 (Cary, NC). Covariance parameter estimates: Location (year), 0.01419 ± 0.00614; Residual, 0.0916 ± 0.00546; Fit statistics—BIC = 922.24; −2 Res log likelihood = 917.48. Benchmark: ^†^ Breeding season pregnancy rate = 90%; Prebreeding BW (%) = 65% pf mature BW; Pre-calving BW = 85% of mature BW. Calculations: % Puberty at the beginning of breeding season = Number of heifers cycling/Total number of heifers. % Pregnancy rate = Number of heifers pregnant/total number. Note: Only heifer calves born in the first 21 of days of the calving season were selected for the study.

## Data Availability

The data presented in this study are available on reasonable request from the corresponding author.

## Supplementary Materials

The following are available online at https://www.mdpi.com/article/10.3390/ani11102800/s1, Table S1: Hay analysis 1, Table S2: Hay analysis 2, Table S3: Mean body weight (kg) at weaning and at breeding, and average daily gain for dam heifers across locations, Table S4: Mean body weight (kg) at weaning and at breeding, and average daily gain for dam heifers across years.
