# Supplementary material for: Difference in Body Weight at Breeding Affects Reproductive Performance in Replacement Beef Heifers and Carries Consequences to Next Generation Heifers
Source: Animals (Basel). 2021 Sep 26;11(10):2800. doi: 10.3390/ani11102800 (PMC8533008; doi:10.3390/ani11102800)
Supplement: Supplementary file 1 [file animals-11-02800-s001.zip › animals-1315116-Supplementary.pdf]

animals

MDPI

Supplementary Materials: Difference in Body Weight at Breeding Affects Reproductive Performance in Replacement Beef Heifers and Carries Consequences in Next Generation Heifers

Ramanathan Kasimanickam [1,*], Vanmathy Kasimanickam [1,2] and Madison McCann [1]

[1] Department of Veterinary Clinical Sciences, College of Veterinary Medicine, Washington State University, Pullman, WA 99164, USA; vkasiman@wsu.edu (V.K.); madison.mccann@wsu.edu (M.M.)
[2] AARVEE Animal Biotech LLC, Corvallis, OR 97333, USA
* Correspondence: ramkasi@wsu.edu

Table S1. Hay analysis 1.

Contents	As Received	100% DM
Moisture, %	9.4	-
Dry Matter, %	90.6	-
Crude Protein, %	9.1	10.0
Acid Detergent Fiber, %	34.2	37.7
Crude Fat, %	1.1	1.2
Ash, %	9.7	10.7
Total Digestible Nutrients, % TDN	54.9	60.6
Net Energy, Maint., Mcal/lb	0.54	0.60
Net Energy, Gain, Mcal/lb	0.31	0.34
Net Energy, Lact., Mcal/lb	0.56	0.62
Digestible Energy, Mcal/lb	1.10	1.21
Met. Energy, Beef, Mcal/lb	0.90	1.00

Table S2. Hay analysis 2.

Contents	As Received	100% DM
Moisture, %	8.7	-
Dry Matter, %	91.3	-
Crude Protein, %	9.8	10.7
Acid Detergent Fiber, %	30.4	33.3
Crude Fat, %	1.3	1.5
Ash, %	9.0	9.9
Total Digestible Nutrients, % TDN	59.4	65.1
Net Energy, Maint., Mcal/lb	0.61	0.67
Net Energy, Gain, Mcal/lb	0.37	0.40
Net Energy, Lact., Mcal/lb	0.61	0.67
Digestible Energy, Mcal/lb	1.19	1.30
Met. Energy, Beef, Mcal/lb	0.98	1.07

Table S3. Mean body weight (kg) at weaning and at breeding, and average daily gain for dam heifers across locations.

Location	Weight Group (MBW*)	*n*	Weight at Weaning, kg	Weigh at Breeding, kg	ADG, kg
Location 1	55	195	235	299	0.8
	65	188	233	352	1.5
Location 2	55	98	236	305	0.9
	65	91	236	349	1.4
Location 3	55	88	238	307	0.9
	65	92	235	351	1.5
Location 4	55	211	237	308	0.9
	65	202	236	346	1.4
Location 5	55	114	235	306	0.9
	65	102	235	350	1.4
Location 6	55	79	236	309	0.9
	65	69	233	345	1.4
Location 7	55	154	237	308	0.9
	65	159	236	354	1.5
Location 8	55	94	232	306	0.9
	65	97	232	349	1.5
Location 9	55	132	238	303	0.8
	65	128	234	347	1.4
Location 10	55	110	235	304	0.9
	65	102	233	351	1.5
Location 11	55	121	238	303	0.8
	65	112	235	347	1.4
Location 12	55	114	236	304	0.9
	65	118	233	350	1.5
Total	55	112	236	305	0.9
	65	118	234	349	1.4

* Mature body weight, 545 kg.

Table S4. Mean body weight (kg) at weaning and at breeding, and average daily gain for dam heifers across years.

Year	Weight Group, (MBW *)	Weight at Weaning, kg	Weigh at Breeding, kg	ADG, kg
2015	55	236	304	0.9
	65	232	347	1.4
2016	55	234	301	0.8
	65	234	349	1.4
2017	55	237	305	0.9
	65	236	350	1.4
2018	55	238	307	0.9
	65	236	351	1.4
2019	55	236	308	0.9
	65	234	348	1.4
Total	55	236	305	0.9
	65	234	349	1.4

* Mature body weight, 545 kg.
